# The American Medical Association

**Published:** 1860-03

**Authors:** Stephen G. Hubbard

**Affiliations:** Secretary; New Haven, Ct.


THE AMERICAN MEDICAL ASSOCIATION will hold its thirteenth annual meeting at New Haven, on the *first Tuesday of June*, 1860.

The secretaries of local societies, colleges, and hospitals are requested to forward the names of delegates, as soon as they are appointed, to

STEPHEN G. HUBBARD, M.D.,
*Secretary.*

NEW HAVEN, CT.

